# Vibrationally Induced Resonances in Lasing

**DOI:** 10.1021/acs.jpclett.5c04028

**Published:** 2026-04-23

**Authors:** Kai Müller, Kimmo Luoma, Christian Schäfer

**Affiliations:** † Institut für Theoretische Physik, 9169 Technische Universität Dresden, D-01062 Dresden, Germany; ‡ Department of Physics and Astronomy, 8058 University of Turku, 20014 Turku, Finland; § Institute of Applied Physics, 27259 TU Wien, Wiedner Hauptstraße 8-10/134, 1040 Vienna, Austria

## Abstract

Optical circuits and light sources, such as lasers, undergo continuous miniaturization. In its extreme, nanolasers might be composed of only a few molecules confined in plasmonic nanoresonators. Few-emitter lasers promise low-energy requirements and fast responses in a footprint that can be inserted into any device or biological tissue. Utilizing the recently developed stacked hierarchy approach, informed from first principles, we demonstrate the impact of the vibrational structure on lasing, using the example of few-molecule lasing in plasmonic cavities. Explicitly accounting for the entire vibrational manifold unveils resonances in the laser intensity that depend on the Stokes shift, drive strength, and number of emitters. Our work identifies the limits of the omnipresent ”incoherent drive” approximation and paves the way for the understanding of nanolasers at the molecular scale.

## Graphical abstract

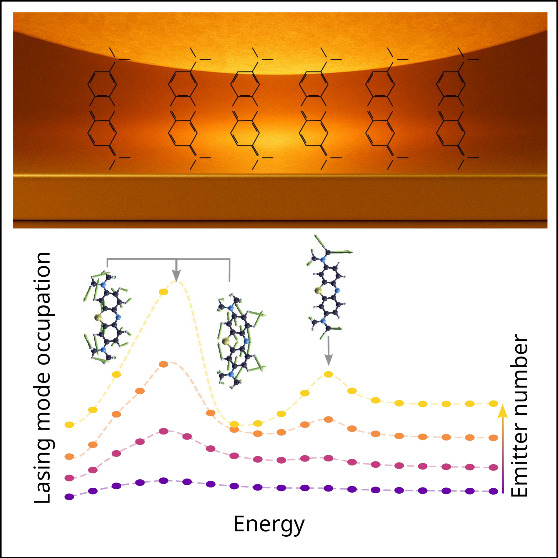

Moore’s law has dominated the development of electronic components for many decades. The continuous shrinking of transistors increased the portability and flexibility of our technical devices while capping power usage and boosting performance. A similar development can be observed for lasers.^2–8^ Lasing occurs when stimulated emission into a common optical mode overcomes all losses, a process that is commonly linked to a macroscopic gain medium but can be sized down to the nanometer scale. Nanolasers come in various shapes, whether it be arrays of plasmonic nanoparticles embedded in a gain medium^9^ or a couple of emitters confined in optical modes with an extension of a few nanometers.^10,11^ Developing a microscopic understanding of such systems can be challenging, especially when the required inversion of occupation necessitates relaxation via the vibrational manifold. For a faithful description, the many-body problem of multiple emitters has to be combined with the influence of non-Markovian vibrational baths.^12–14^ Inside a cavity, this leads to a combination of large Hilbert spaces, all-to-all interactions, and non-Markovian evolution that provides a significant barrier for most methods and encourages the use of simplified or effective models. Common approximations include the Holstein–Tavis–Cummings model,^15–18^ where the vibrational spectrum is replaced by a single damped mode, or the combination of coherent drive and vibrational relaxation into an effective incoherent drive,^10,19,20^ assuming fast decay via a structureless vibrational bath.

In this letter, we provide a detailed theoretical discussion of molecular few-emitter lasing that is largely informed from first principles and accounts for the coherent excitation, inversion, and lasing process without any of the approximations mentioned. We achieve this by utilizing the newly developed stacked BBGKY–HEOM hierarchy, which we present in ref 1. The incorporation of the entire vibrational manifold for each emitter results in noticeable deviations from paradigmatic approximations and unveils resonant enhancements of the lasing process that are absent in a description that utilizes the widely used approximation of incoherent drives.^19^

*Molecular Lasing Informed from First Principles*: We postulate the existence of vibrationally induced resonances in few-emitter lasing, motivated by a recent experimental realization. The underlying physics as well as the resulting observable signatures are transferable to a wide range of molecular gain materials. Figure 1a illustrates the system under investigation: a small ensemble (*N* ≈ 2–20) of methylene blue (MB) molecules embedded in a plasmonic nanocavity formed by a gold nanoparticle positioned above a metallic mirror.^10,11^ The locally enhanced fields in the plasmonic nanocavity are dominated by dipolar Mie modes with a longitudinal character. A simplified description of those modes as purely bilinear is reasonable as long as the molecular electronic structure does not influence the plasmonic excitation (we refer the interested reader to the ever increasing literature on quadratic corrections for transversal fields^21,22^ and also point out that longitudinal plasmonic excitations will be screened, thus renormalized, when the surface of the nanoplasmonic particle is considerably occupied with molecules^23^). We therefore start from a Hamiltonian of the form

1 $$H=\overset{N}{\underset{i=1}{\sum }}H_{\mathrm{mol}}^{\left(i\right)}+H_{\mathrm{drive}}+\underset{i}{\sum }g_{\mathrm{cav}}\left(L_{i}a^{†}+{L_{i}}^{†}a\right)+\omega_{\mathrm{cav}}a^{†}a$$

where *a* (*a* ^†^) is the annihilation (creation) operator of the cavity mode and *L*_*i*_ is the coupling operator for molecule *i*. *H*_drive_ describes the coherent external drive and *H*_mol_^(*i*)^ is the local Hamiltonian of molecule *i*. Aggregation and the direct dipole–dipole interaction are largely avoided in the experiment by encapsulating the molecules in dielectric cucurbit[7]­uril hosts.^10,11^ In eq 1, we have assumed that all molecules couple equally to the cavity mode. While this is not the case in typical experiments, it simplifies the numerical treatment, and we argue later that an inhomogeneous coupling strength will not qualitatively affect our results. For each individual molecule, we restrict the electronic degrees of freedom to the many-body states associated with the first bright electronic transition ω̃_0_. However, we take the full spectrum of vibrational modes with frequencies ω_λ_^vib^ and Huang–Rhys factors *S*_λ_ into account. For a realistic vibrational spectrum, electronic and vibrational frequencies as well as Huang–Rhys factors are estimated using the time-dependent density functional theory^24–26^ for the methylene blue molecule used in the experiment^10^ (see the ). In the double-harmonic Franck–Condon approximation for the electron–phonon coupling, each of the 107 vibrational normal modes is described as a harmonic oscillator, where the equilibrium position of the oscillator is shifted by a displacement $\Delta_{\lambda }=\sqrt{2S_{\lambda }/\omega_{\lambda }}$ if the molecule is in the electronically excited state. Within this approximation, the first part of *H*_mol_^(*i*)^ = *H*_m_^(*i*)^ + *H*_mE_^(*i*)^ takes the form

2 $$\begin{array}{c} H_{m}^{\left(i\right)}=\frac{{\mathrm{\omega ̃}}_{0}}{2}\sigma_{i}^{z}+\overset{107}{\underset{\lambda =1}{\sum }}\frac{{p_{i,\lambda }}^{2}}{2}+\frac{{\left(\omega_{\lambda }^{\mathrm{vib}}\right)}^{2}}{2}{\left(q_{i,\lambda }-\Delta_{\lambda }\sigma_{i}^{+}\sigma_{i}^{-}\right)}^{2}\\ =\frac{\omega_{0}}{2}\sigma_{i}^{z}-\frac{1}{\sqrt{2}}\overset{107}{\underset{\lambda =1}{\sum }}\omega_{\lambda }^{\mathrm{vib}}\sqrt{S_{\lambda }}\sigma_{i}^{+}\sigma_{i}^{-}\left(b_{i,\lambda }+{b_{i,\lambda }}^{†}\right)+\overset{107}{\underset{\lambda =1}{\sum }}\omega_{\lambda }^{\mathrm{vib}}{b_{i,\lambda }}^{†}b_{i,\lambda } \end{array}$$

where *q*_*i*,λ_ and *p*_*i*,λ_ are the mass renormalized conjugate position and momenta of the λth normal mode of molecule *i*(see the for a detailed derivation). The operators *b*_λ_ (*b*_λ_^†^) are the corresponding annihilation (creation) operators, with $b_{\lambda }=\sqrt{\omega_{\lambda }/2}\left(q_{\lambda }+ip_{\lambda }/\omega_{\lambda }\right)$ . Furthermore, we have introduced the vertical Franck–Condon excitation energy ω_0_ = ω̃_0_ + ∑_λ_ω_λ_^2^Δ_λ_^2^/2, where ω_0_ = 20 857 cm^–1^. Due to the embedding of the MB molecules in the surrounding dielectric host medium,^11^ each of these vibrational modes in turn couples to a continuum of environmental modes, which we describe with an ohmic spectral density *J*_E_(ω) (see Figure 1b and the ). These environmental modes and their interaction with the molecule are contained in *H*_mE_^(*i*)^, which forms the second part of the molecular Hamiltonian *H*_mol_^(*i*)^. They lead to a broadening of the vibrational spectrum and ensure that the vibrational modes quickly relax to the normal (shifted) equilibrium position if the molecule is in the ground (excited) state.^27^ Due to the rapid relaxation, the setup resembles a four-level lasing scheme in the sense of Figure 1c. Coherent excitation to state |4⟩ is followed by a fast relaxation into the new equilibrium position of the vibrational modes |3⟩, from which the state decays into |2⟩ via cavity emission. The decay is then again followed by a fast vibrational relaxation into |1⟩. Note that because the state |2⟩ (|3⟩) arises from the shifted equilibrium positions of the vibrational modes, it is generally not orthogonal to |1⟩ (|4⟩).

**Figure 1:**
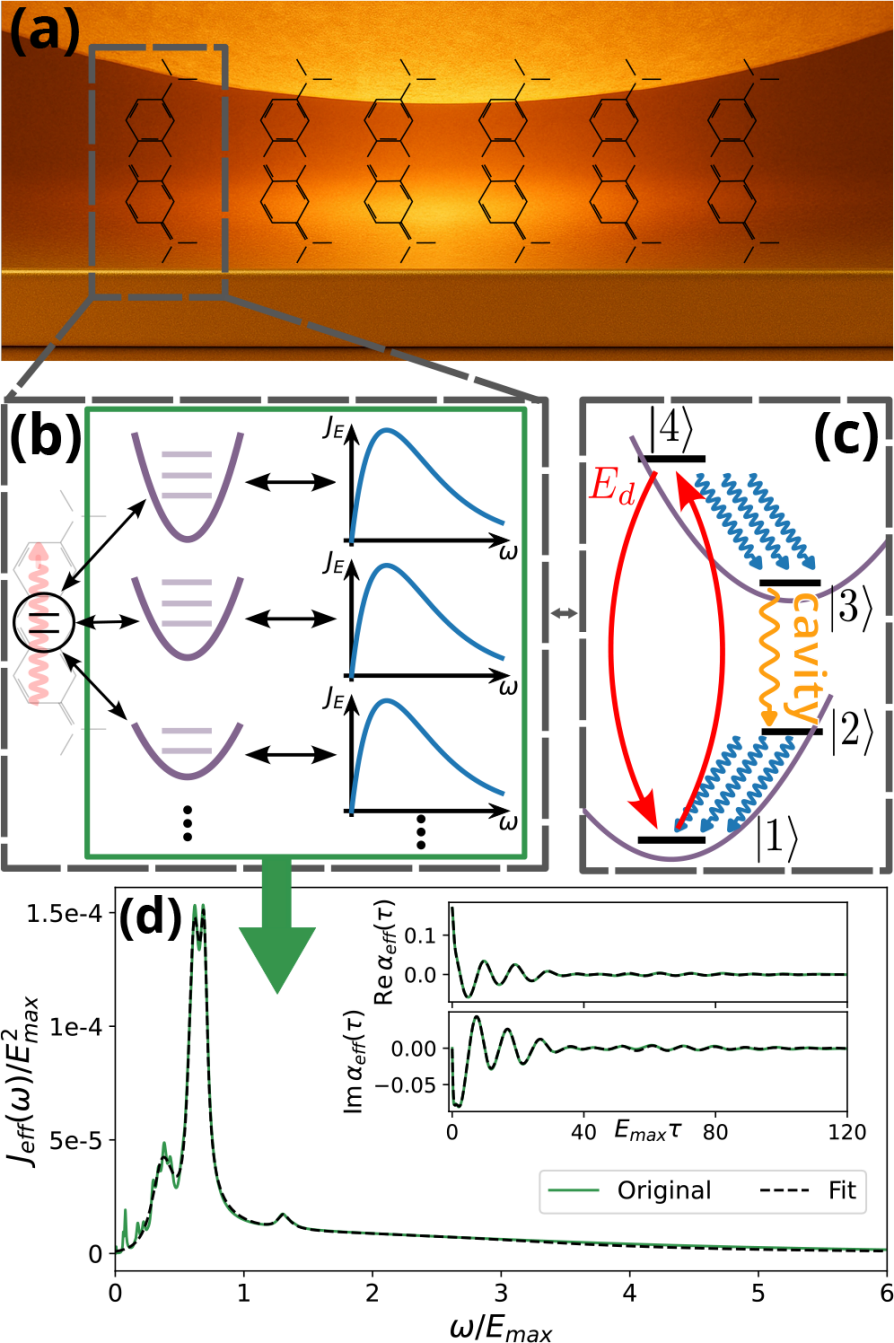
Overview of the few-emitter lasing system. (a) Sketch of the plasmonic nanocavity, comprised of a gold nanoparticle above a gold surface. (b) We restrict our model of the electronic states to the first bright transition, leading to a two-level system. However, we take the full spectrum of vibrational modes into account, which we approximate as a large set of harmonic oscillators (purple). Each oscillator in turn couples to a continuum of vibrational modes that we model with an ohmic spectral density (blue). All of the vibrations can be combined into a single effective bath with the spectral density *J*_eff_(ω) shown in panel d (green solid line). The corresponding bath correlation function shown in the inset is then fitted with exponentials (black dashed line). Panel c shows a sketch of the lasing mechanism, where the states |2⟩ and |3⟩ are distinguished from the states |1⟩ and |4⟩ by a different vibrational state. Purple parabolas in the background indicate the shifted harmonic potential surfaces.

For an exact treatment of this process, we proceed by combining all 107 vibrational modes and their environments in an exact way into a single bath with effective spectral density^28^ *J*_eff_(ω) = ∑_λ_|*g*_λ_^eff^|^2^δ­(ω – ω_λ_) shown in Figure 1d. This process is performed for each molecule and is described in detail in the .

The (*N*) molecules are driven by a coherent field with amplitude 2*E*_d_ and frequency ω_0_ according to *H*_drive_ = 2*E*_d_ cos­(ω_0_ *t*)∑_*i*_σ_*i*_^*x*^. In an interaction picture that rotates with the drive frequency ω_0_, we can perform a rotating wave approximation, assuming that ω_0_ ≫ *E*_d_,*g*_cav_ (see the ). The resulting total Hamiltonian in the interaction picture *H* ^I^ then takes the form

3 $$H^{I}=\underset{j}{\sum }\left(E_{d}\sigma_{x}^{j}+\underset{\lambda }{\sum }\omega_{\lambda }^{\mathrm{eff}}{b_{\lambda ,j}}^{†}b_{\lambda ,j}-\underset{\lambda }{\sum }g_{\lambda }^{\mathrm{eff}}\sigma_{+}^{j}\sigma_{-}^{j}\left({b_{\lambda ,j}}^{†}+b_{\lambda ,j}\right)\right)+g_{\mathrm{cav}}\underset{j}{\sum }\left(\sigma_{j}^{+}a+\sigma_{j}^{-}a^{†}\right)+\left(\omega_{\mathrm{cav}}-\omega_{0}\right)a^{†}a$$

We include the effects of cavity loss and spontaneous emission by means of the GKSL master equation^29,30^ for the total state ρ_tot_

4 $${\mathrm{\rho ̇}}_{\mathrm{tot}}=-i\left[H^{I},\rho_{\mathrm{tot}}\right]+\Gamma_{↓}\underset{j}{\sum }D\left[\sigma_{-}^{j}\right]\left(\rho_{\mathrm{tot}}\right)+\kappa D\left[a\right]\left(\rho_{\mathrm{tot}}\right)$$

with $D\left[L\right]\left(\rho \right)=2L\rho L^{†}-\left\{L^{†}L,\rho \right\}$ To solve eq 4, we utilize the recently developed BBGKY–HEOM approach,^1^ based on a combination of the hierarchical equations of motion (HEOM) and the Bogoliubov–Born–Green–Kirkwood–Yvon (BBGKY) hierarchy. The method allows for an efficient, approximate description of many-body systems interacting with local or global non-Markovian baths. We truncate the BBGKY hierarchy by neglecting three-body correlations beyond a Gaussian approximation. As a result, the equations of motion of the system become independent of the number of particles *N*. The HEOM then provides an in principle exact treatment of the non-Markovian baths after their bath correlation function was fitted with exponentials. The details of the method can be found in an accompanying article,^1^ along with extensive benchmarks for different systems. Here, we proceed to show the application of the BBGKY–HEOM method to eq 4 and the physical results.

The fit of the bath correlation function α_eff_(τ) = ∫_0_^∞^ *J*_eff_(ω)​e^–*i*ω*t*^ dω with a sum of 5 exponentials as α_eff_(τ) ≈ ∑_*i*_ *G*_*i*_ exp­(−*W*_*i*_τ) with $G_{i},W_{i}\in C$ is shown in Figure 1d. We treat the cavity as an additional global bath, coupling to all molecules. Since the bath correlation function of the cavity α_cav_(τ) = ⟨*a*(*t*)*a* ^†^(*s*)⟩ = *g*_cav_^2^e^–*i*ω_cav_τ–κ|τ|^ is already exponential, no additional fit is required. The full evolution equation for BBGKY–HEOM can be found in the .

We now propagate the state of the emitters with different drive strengths *E*_d_, up to a maximum drive strength *E*_max_ = 0.1ω_0_, and cavity parameters *g*_cav_ = 0.2*E*_max_ and κ = 3.3*E*_max_ until the respective steady states are reached. The results are shown in Figure 2a and b, where we show the occupation of the excited electronic state *p*_e_ and the cavity in the steady state for 10 molecules and different coherent driving strengths *E*_d_ (magenta circles). Note that the intensity of the laser is proportional to the cavity occupation, *I* = κ⟨*a* ^†^ *a*⟩. The results are contrasted with a second set of BBGKY–HEOM simulations (black crosses), where the coherent drive and the vibrational bath have been combined into an effective incoherent pumping, such that the molecules evolve according to

5 $$\begin{array}{c} {\mathrm{\rho ̇}}_{\mathrm{incoh}}=-i\left[H_{\mathrm{incoh}}^{I},\rho_{\mathrm{incoh}}\right]+\Gamma_{↓}\underset{j}{\sum }D\left[\sigma_{-}^{j}\right]\left(\rho_{\mathrm{incoh}}\right)+E_{d}\underset{j}{\sum }D\left[\sigma_{+}^{j}\right]\left(\rho_{\mathrm{incoh}}\right)+\kappa D\left[a\right]\left(\rho_{\mathrm{incoh}}\right)\\ H_{\mathrm{incoh}}^{I}=g_{\mathrm{cav}}\left(\underset{j}{\sum }\sigma_{+}^{j}a+\underset{j}{\sum }\sigma_{-}^{j}a^{†}\right)+\left(\omega_{\mathrm{cav}}-\omega_{0}\right)a^{†}a \end{array}$$

How accurate this approximation is will depend heavily on the coupled bath, i.e., on the molecular and host structure. Additionally, note that this comparison is supposed to be of a qualitative nature, as the different steady states for a coherent pumping with strength *E*_d_ are not directly comparable to those for incoherent pumping with strength *E*_d_. Instead, we focus our analysis on the qualitative behavior as the driving strength is increased.

**Figure 2:**
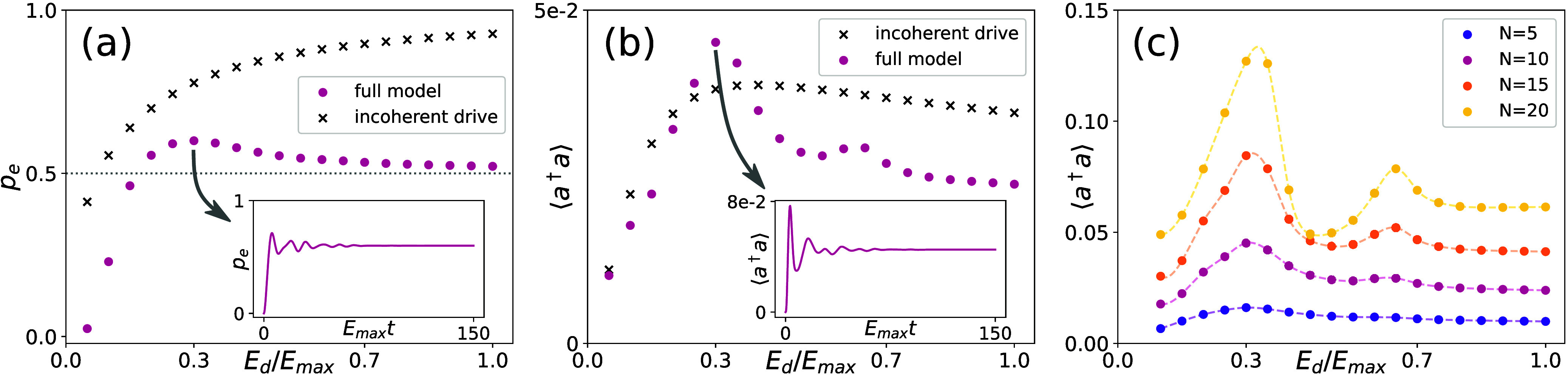
Vibrational impact on few-emitter lasing. We compare the qualitative steady-state behavior for increasing the coherent drive in the full model (eqs 3 and 4) against an effective incoherent drive that neglects the vibrational structure (eq 5). Panels a and b show the occupation of the excited electronic state, *p*_e_, and the cavity mode, *a* ^†^ *a*, respectively, for a plasmonic cavity containing 10 molecules. Both panels show maxima arising from resonances with the vibrational spectrum that are missed by effective treatment with an incoherent drive. Those maxima become more pronounced for increasing *N* as shown in panel c.

Figure 2a demonstrates the population inversion required for a lasing transition. In contrast to the predictions from the incoherent drive, the full model does not indefinitely increase its inversion but rather reaches a plateau at large driving strengths. There, the coherent drive leads to a fast polarization that dominates the dynamics, resulting in *p*_e_ ∼ 0.5. Interestingly, we find a maximal inversion at *E*_d_/*E*_max_ ≈ 0.3, with the corresponding time evolution of *p*_e_ shown in the inset. The steady-state cavity occupation in Figure 2b features distinct maxima in addition to a background that is reminiscent of the solution using an incoherent drive. These maxima become even more pronounced for larger numbers of molecules *N*, as they are enhanced by the self-amplifying lasing process (see Figure 2c). This enhancement can be partially understood as a collective enhancement ⟨*a* ^†^ *a*⟩ ∼ *N* ^2^. However, also, the relative strength between peaks and the background increases with *N* due to correlated dynamics that extend beyond the mean-field theory. We provide a more detailed discussion of this effect in the .

To investigate the origin of these maxima, it is instructive to compare their location to the vibrational spectrum *J*_eff_(ω), as shown in Figure 3, for the same parameters as in Figure 2 with *N* = 5. We find that the maxima line up with peaks in the effective vibrational spectrum and occur if the drive strength in atomic units *E*_d_ is chosen to be half of the peak frequency (solid lighter line).

**Figure 3:**
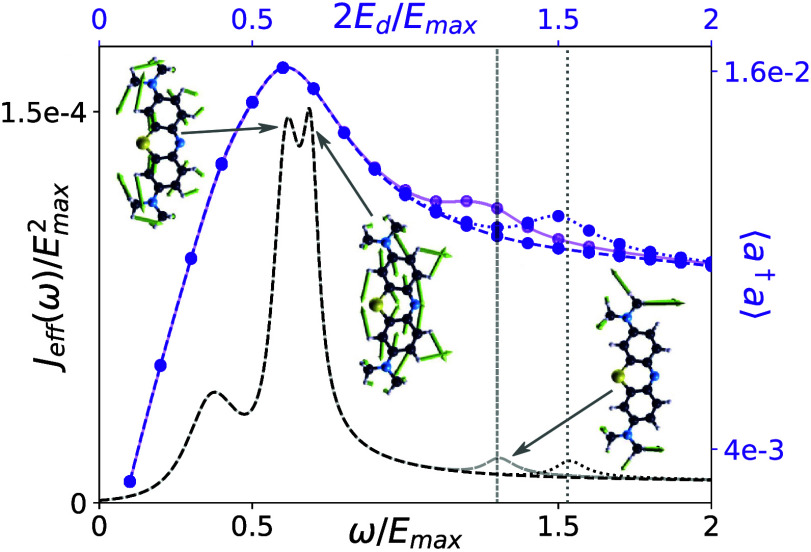
Vibrationally induced resonances. Steady-state cavity occupation for *N* = 5 (purple) and effective vibrational spectrum are shown on a shared *x* axis, with 2*E*_d_ = ω. Maxima in ⟨*a* ^†^ *a*⟩ line up with peaks in the spectral density. Solid, lighter lines correspond to the data in Figures 2c and 1d, respectively. Intentionally omitting (shifting) a peak in *J*_eff_(ω) also removes (shifts) the corresponding occupation maxima, as evidenced by the darker dashed (dotted) lines. Insets show dominant vibrational modes at the peak positions.

Artificially removing a peak in the spectral density, e.g., at ω/*E*_max_ ≈ 1.3, we find that also the associated maximum in the cavity occupation vanishes (darker, dashed lines), and shifting the peak also shifts the corresponding maxima (darker, dotted lines). We thus attribute this effect to resonances between the driving strength strength and the effective vibrational spectrum *J*_eff_. Insets in Figure 3 illustrate the dominant vibrational modes. The occurrence of resonances at ω = 2*E*_d_ can be rationalized from a simplified model of a single dissipation-less vibrational mode for a molecule under resonant drive *H*_simple_ = *E*_d_σ_*x*_ + *g* ^vib^σ_+_σ_–_(*b* + *b* ^†^) + ω^vib^ *b* ^†^ *b*. Up to a 90° rotation *x* → *z* and *z* → −*x* and a shift of the oscillators, this model is equivalent to the quantum Rabi model,^31^ which also features a resonance at ω = 2*E*_d_. In our case, this resonance leads to an increased occupation of the vibrational modes, akin to an enhanced occupation of the state |3⟩ in Figure 2c and, thus, facilitates the lasing process. Intuitively, driving the molecular system results in an AC Stark effect that splits the original states. If the energies of neighboring vibrational states align, which happens precisely at ω = 2*E*_d_, energy transfer, vibrational occupation, and thus lasing are maximized. We provide a more detailed discussion in of the .

It is important to note that the resonances are dictated by the peaks in the effective vibrational spectrum *J*_eff_. Thus, their exact position and height are influenced by the spectral density of the environmental modes *J*_E_(ω) and can in general differ from the frequencies of the isolated modes ω_λ_^vib^ in eq 2. Furthermore, since *J*_eff_ does not depend on the cavity coupling, we expect that our results still hold for inhomogeneous cavity couplings. The many-body picture employed in this section, therefore, provides straightforward access to vibronic coupling and recommends itself as a natural basis to describe few-emitter lasing.

*Conclusion*. Few-emitter lasers, here molecules in a plasmonic nanocavity, are intricate systems in which optical, thermal, and chemical processes compete. Fostering this young domain requires a comprehensive understanding at the molecular level and, thus, a holistic description. Instead of combining coherent drive and vibrational relaxation into an effective incoherent drive, we have moved beyond this paradigmatic approximation and accounted for the entire vibrational manifold. Our approach is largely informed from first principles and based on the recently developed stacked BBGKY–HEOM hierarchy.^1^

We observe two major differences from the simplified incoherent model. First, maintaining inversion at a larger drive strength is challenged by the limited vibrational relaxation speed compared to ever increasing polarization frequencies of the drive transition. Second and especially surprising, we identify clear resonances in the inversion and mode occupation for specific combinations of the driving strength and vibrational frequency. The resonances are induced when AC Stark shifts energetically align the Franck–Condon transition with neighboring vibrational states, populating the vibrational mode and enhancing the lasing transition. Those resonant features increase with the number of emitter molecules and become substantial in the double-digit domain. Few-emitter lasing is thus not only more intricate than widely believed but also offers a path to count the number of coherently coupled molecules in cavity environments.

The strong confinement of molecules in a domain of only a few nanometers suggests that direct dipole–dipole interactions (despite the embedding), multimode coupling, vibrational heating,^32^ or even charge transfer between nanocavity and emitter^33–35^ could further complicate a faithful description. Our current level of theory therefore provides a realistic yet simplified description of molecular few-emitter lasing, and our discussion can be transferred to various realizations of a similar design. Extensions to the theory can be incorporated into this approach and will be the subject of future studies.

## Supplementary Material





## Notes

Derivation of the effective spectral density based on density functional theory, comparison for the absorption spectrum to ORCA, derivation of the Hamiltonian in the interaction picture, and numerical details (PDF)
Transparent Peer Review report available (PDF)
