# Supplementary material for: Vibrationally Induced Resonances in Lasing
Source: J Phys Chem Lett. 2026 Apr 23;17(18):5275–9. doi: 10.1021/acs.jpclett.5c04028 (PMC13158989; doi:10.1021/acs.jpclett.5c04028)
Supplement: Supplementary file 1 [file jz5c04028_si_001.pdf]

# Vibrationally Induced Resonances in Lasing

Kai Müller,[1] Kimmo Luoma,[2] and Christian Schäfer[3,*]

[1]*Institut für Theoretische Physik, Technische Universität Dresden, D-01062 Dresden, Germany.*
[2]*Department of Physics and Astronomy, University of Turku, 20014 Turku, Finland.*
[3]*Institute of Applied Physics, TU Wien, Wiedner Hauptstrasse 8-10/134, Vienna, 1040, Austria.*

**Contents**

**S1. Derivation of the few emitter lasing Hamiltonian** 1
A. Huang-Rhys factors from TDDFT 1
B. Effective spectral density of intra-molecular vibrations 2
C. Interaction picture dynamics for few emitter lasing 3

**S2. Additional simulation data** 5
A. Rescaled coupling strength 5
B. Vibrational occupation 5
C. Off-resonant driving 6

**S3. Summary of the BBGKY-HEOM method** 6

**S4. Numerical Details** 7

**References** 8

## S1. DERIVATION OF THE FEW EMITTER LASING HAMILTONIAN

### A. Huang-Rhys factors from TDDFT

All (TD)DFT calculations have been performed with the ORCA code [1] using the PBE exchange-correlation potential [2] with Grimme D3 dispersion correction [3] and def2-TZVPD basis. We expect the (electronic) structure of methylene-blue (MB) molecules to depend heavily on the embedding in cucurbit[7]uril and in the presence of the nanoparticle-on-mirror set-up. For simplicity, we have assumed here the oxidized form (positively charged $MB^+$) of a single MB molecules, which is typically associated with an intense blue color.

First, the ground-state geometry was converged, and electronic absorption spectra were calculated using the Casida-TDDFT approach (employing the Tamm-Dancoff approximation). Second, the geometry of the S1 and S2 states have been relaxed. We found the S1 state to be virtually dark for absorption and emission (with a Stokes shift of 0.269 eV) and have therefore decided to focus on the S2 transition (Stokes shift 0.119 eV) for subsequent discussion. We note that the selected level of theory is insufficient for a direct comparison with experimental spectra as Tamm-Dancoff approximation, charge state, and the encapsulation can be expected to result in noticeable spectral shifts. Using higher-level exchange-correlation potentials (B3LYP+D4) and a continuous polarization model for water flips the excitation order (S1 is then bright) but leads otherwise to minor changes in the excitation frequency (486.8 nm) and oscillator strength at a considerably increased cost. The Tamm-Dancoff approximation has, on the other hand, a notable impact. Full Casida-TDDFT shifts the bright absorption (S1 now) to 550.5 nm and future studies that aim at full theoretical consistency with the experiment are therefore advised to avoid the Tamm-Dancoff approximation.

In the following we give a brief derivation of Eq. (2) and show how the coupling strengths and frequencies of the vibrational modes are obtained from the DFT calculations. We express the nuclear coordinates through the deviations $\boldsymbol{\eta}$ from their respective equilibrium positions. In the harmonic approximation the (classical) Hamiltonian governing

*Electronic address: christian.schaefer@tuwien.ac.at

their dynamics takes the form of coupled harmonic oscillators with the mass matrix $\mathbf{M}$ and the Hessian Matrix of the potential energy $\mathbf{K}$ (obtained from the DFT calculations). It reads

$$\begin{aligned} H =& \frac{1}{2}\dot{\boldsymbol{\eta}}^T\mathbf{M}\dot{\boldsymbol{\eta}} + \frac{1}{2}\boldsymbol{\eta}^T\mathbf{K}\boldsymbol{\eta}, \\ =& \frac{1}{2}\dot{\boldsymbol{\mu}}^2 + \frac{1}{2}\boldsymbol{\mu}^T\mathbf{M}^{-1/2}\mathbf{K}\mathbf{M}^{-1/2}\boldsymbol{\mu}, \\ \boldsymbol{\mu} =& \mathbf{M}^{1/2}\boldsymbol{\eta}. \end{aligned} \tag{S1}$$

The mass re-normalized vibrational eigenmodes $u_k$ and the corresponding frequencies $\omega_l$ are found by means of diagonalization

$$\left[\mathbf{M}^{-1/2}\mathbf{K}\mathbf{M}^{-1/2}\right]\mathbf{u}_k = \omega_k^2\mathbf{u}_k. \tag{S2}$$

In the electronic groundstate the vibrations are decoupled in the eigenbasis

$$\begin{aligned} x_k =& \boldsymbol{\mu}\cdot\mathbf{u}_k, \\ H =& \frac{1}{2}\sum_k \dot{x}_k^2 + \omega_k^2 x_k^2 \end{aligned} \tag{S3}$$

and can be canonically quantized

$$\hat{H} = \sum_k \frac{\hat{p}_k}{2} + \frac{\omega_k^2}{2}\hat{q}_k^2. \tag{S4}$$

In the double harmonic approximation the dynamics in electronically excited state are governed by the same frequencies but shifted equilibrium positions $\boldsymbol{\mu} \to \boldsymbol{\mu} - \boldsymbol{\Delta}$, where $\boldsymbol{\Delta}$ is the mass weighted nuclear displacement between absorption (at 479.5 nm) and emission (at 502.5 nm) $\boldsymbol{\Delta} = \mathbf{M}^{1/2}(\mathbf{R}_{S0} - \mathbf{R}_{S2})$. Along the $k$-th eigenmode we then obtain a displacement of $\Delta_k = \boldsymbol{\Delta}\cdot\mathbf{u}_k$ in the excited state, leading to the Hamiltonian $H_m$ (Eq. (2) in the letter). The Huang-Rhys factors defined as $S_k = \omega_k\Delta_k^2/2$ are thus obtained as

$$S_k = \frac{\omega_k}{2}((\mathbf{R}_{S0} - \mathbf{R}_{S2})\cdot\sqrt{\mathbf{M}}\cdot\mathbf{u}_k)^2. \tag{S5}$$

### B. Effective spectral density of intra-molecular vibrations

Eq. (2) describes an isolated molecule, where the vibrations form the stick spectrum shown in Fig. S1. To account for the dielectric cage and solvate, in which the molecules are embedded, we couple each molecular vibration to an (accoustic) phonon bath with an ohmic spectral density $J_E(\omega)$. It is important to note that the phonon bath will always relax the vibrational modes to their respective groundstate, which is shifted if the molecule is electronically excited. To ensure this, we describe the coupling between the molecular vibrations and the environmental phonon modes with the coupling operator $b_\lambda - \Delta_\lambda\sigma_+\sigma_-$ [4]. The influence of the phonon modes is captured in $H_{mE}$. The total Hamiltonian for a single molecule and the phonon bath with annihilation operators $d_\xi$ now reads

$$\begin{aligned} H_{mol} =& H_m + H_{mE}, \\ H_m =& \frac{\omega_0}{2}\sigma^z - \frac{1}{\sqrt{2}}\sum_{\lambda=1}^{107}\omega_\lambda^{vib}\sqrt{S_\lambda}\sigma_+\sigma_-(b_\lambda + b_\lambda^\dagger) + \sum_{\lambda=1}^{107}\omega_\lambda^{vib}b_\lambda^\dagger b_\lambda, \\ H_{mE} =& \sum_{\lambda,\xi}\omega_\xi d_{\lambda,\xi}^\dagger d_{\lambda,\xi} + \sum_{\lambda,\xi}\left(g_\xi(b_\lambda - \Delta_\lambda\sigma_+\sigma_-)d_{\lambda,\xi}^\dagger + \text{h.c.}\right). \end{aligned} \tag{S6}$$

The ohmic spectral density is given as

$$\begin{aligned} J_E(\omega) =& \sum_\xi |g_\xi|^2\delta(\omega - \omega_\xi), \\ =& A\omega\exp(-\omega/\Lambda)\theta(\omega). \end{aligned} \tag{S7}$$

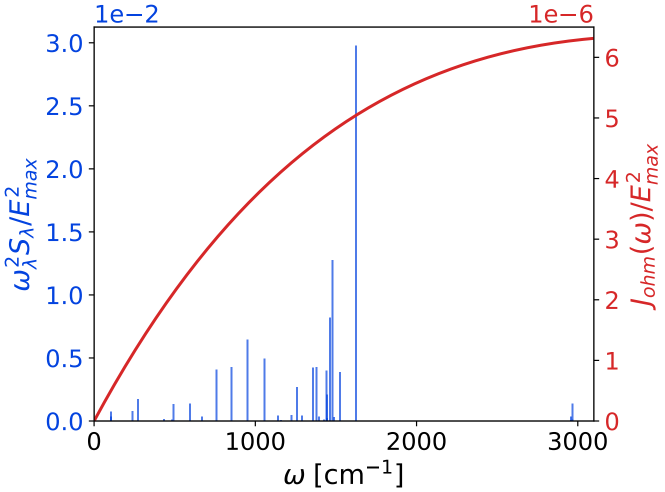


FIG. S1: Squared coupling strengths of the vibrational modes (blue) as obtained from DFT and ohmic spectral density of the phonon environment (red).

The choice of $J_E(\omega)$ is motivated by acoustic phonons [5], where the parameter $A$ scales the total coupling strength and thus the broadening of each peak. To achieve a reasonable broadening we choose $A = 0.025$ cm$^{-2}$ and $\Lambda = 3500$ cm$^{-1}$, leading to the spectral density shown in the background of Fig. S1. Various broadenings are tested and we find minor influence on the results presented in the main manuscript. The cutoff frequency $\Lambda$ is chosen such that the structure of the resulting spectral density $J_{eff}$ (see below) is not changed upon further increase.
Ref. [6] shows how each of the 107 damped vibrational modes can equally be described by an effective spectral density

$$J_{\lambda,ESM}(\omega) = \frac{\Delta_\lambda^2 A\omega^3 e^{-\omega/\Lambda}\theta(\omega)}{\pi^2 A^2\omega^2 e^{-2\omega/\Lambda} + \left(\omega - \omega_\lambda + A\Lambda - A\omega e^{-\omega/\Lambda}\,\mathrm{Ei}(\omega/\Lambda)\right)^2},$$
$$\mathrm{Ei}(x) = -\int_{-x}^{\infty} \frac{e^{-t}}{t}\,\mathrm{d}t\,. \tag{S8}$$

We thus broaden each line in Fig. S2 according to Eq. (S8), which leads us to the effective spectral density seen by the electronic degree of freedom $J_{eff}(\omega)$ shown in Fig. 1(d) and the effective molecular Hamiltonian

$$H_{mol,eff} = \frac{\omega_0}{2}\sigma^z - \sum_\lambda g_\lambda^{eff}\sigma_+\sigma_-(b_\lambda + b_\lambda^\dagger) + \sum_\lambda \omega_\lambda^{eff} b_\lambda^\dagger b_\lambda,$$
$$J_{eff}(\omega) = \sum_\lambda \left|g_\lambda^{eff}\right|^2 \delta(\omega - \omega_\lambda^{eff}). \tag{S9}$$

By construction, the reduced dynamics of the electronic system is identical under Eq. (S9) and Eq. (S6). This allows us to replace to replace $H_{mol}$ by $H_{mol,eff}$ in the following. The spectral density $J_{eff}(\omega)$ was used to parameterize our BBGKY-HEOM model by fitting the corresponding correlation function $\alpha_{eff}(\tau) = \int_0^\infty J_{eff}(\omega)e^{-i\omega t}\,\mathrm{d}\omega$ with a sum of 5 exponentials. The fit was performed with a simple minimization procedure. In Fig. S2 we compare the resulting absorption spectrum of a single molecule to the path-integral implementation of ORCA [7] and observe qualitative agreement, with some quantitative deviations. A quantitative comparison is complicated by the different treatment of linewidth-broadening in ORCA.

### C. Interaction picture dynamics for few emitter lasing

From the description of a single molecule in Eq. (S9) we now move to multiple molecules that couple identically with strength $g_{cav}$ to a common cavity mode that is in resonance with the electronic transition and are subject to a coherent drive with amplitude $2E_d$ and frequency $\omega_d$ as described by Eq. (1) in the main text. As discussed above we

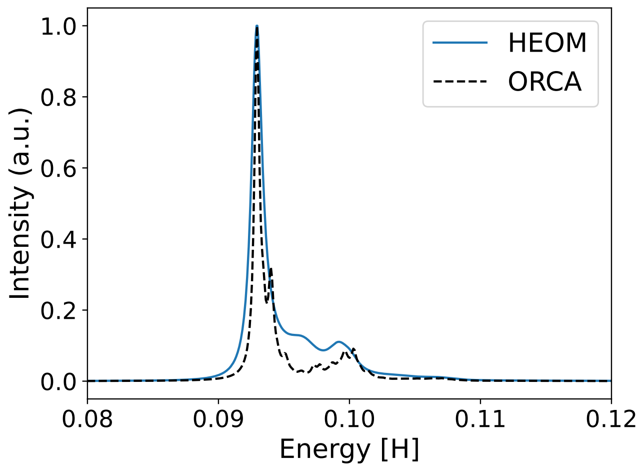


FIG. S2: Absorption spectrum of the molecule based on the Hamiltonian (S9) (blue) compared to the absorption spectrum predicted by ORCA (dashed black). A quantitative comparison is complicated by the different treatment of linewidth-broadening in ORCA.

may replace $H_{mol}^{(j)}$ by $H_{mol,eff}^{(j)}$ without changing the dynamics of the electronic states or the cavity mode, leading to

$$H = \sum_j^N H_{mol,eff}^{(j)} + \sum_j^N 2E_d \cos(\omega_d t)\sigma_j^x + \sum_j g_{cav}\sigma_j^x(a + a^\dagger) + \omega_{cav} a^\dagger a. \tag{S10}$$

Cavity loss and spontaneous decay of the individual molecules are accounted for via a master equation description, such that the total system $\rho_{tot}$ consisting of the molecules and the cavity mode evolves according to

$$\dot{\rho}_{tot} = -i[H, \rho_{tot}] + \kappa\mathcal{D}[a](\rho_{tot}) + \Gamma_\downarrow \sum_i \mathcal{D}[\sigma_-^i](\rho_{tot}). \tag{S11}$$

We now go into an interaction picture $\rho_{tot}^I = U_I \rho U_I^\dagger$ with

$$U_I = \exp\left(i\omega_d t\left(\sum_j \sigma_j^z/2 + a^\dagger a\right)\right), \tag{S12}$$

resulting in the Hamiltonian

$$\begin{aligned} H^I = & \frac{\omega_o - \omega_d}{2}\sum_j \sigma_j^z + E_d \sum_j \sigma_j^x + e^{2i\omega_d t}\sigma_j^+ + e^{-2i\omega_d t}\sigma_j^- \\ & + \sum_j \left(-\sum_\lambda g_\lambda^{eff}\sigma_j^+\sigma_j^-(b_{\lambda,j}^\dagger + b_{\lambda,j}) + \sum_\lambda \omega_\lambda^{eff} b_{\lambda,j}^\dagger b_{\lambda,j}\right) \\ & + g_{cav}\left(\sum_j \sigma_j^+ a + \sum_j \sigma_j^- a^\dagger + \sum_j e^{2i\omega_d t}\sigma_j^+ a^\dagger + \sum_j e^{-2i\omega_d t}\sigma_j^- a\right) \\ & + (\omega_{cav} - \omega_d)a^\dagger a. \end{aligned} \tag{S13}$$

As long as the driving frequency $\omega_d$ is the fastest timescale in the interaction picture Hamiltonian, we can safely discard counter-rotating terms $\propto e^{\pm 2i\omega_d t}$ that oscillate too fast to influence the dynamics of the system. This in particular implies a drive which is near resonant with both the electronic transition as well as the cavity frequency , sucht that $|\omega_0 - \omega_d|$, $|\omega_{cav} - \omega_d| \ll \omega_d$. For a resonant drive $\omega_d = \omega_0$ we end up at Eq. (3) of the main letter. Additional data in for off-resonant driving is shown in Sec. S2 C.

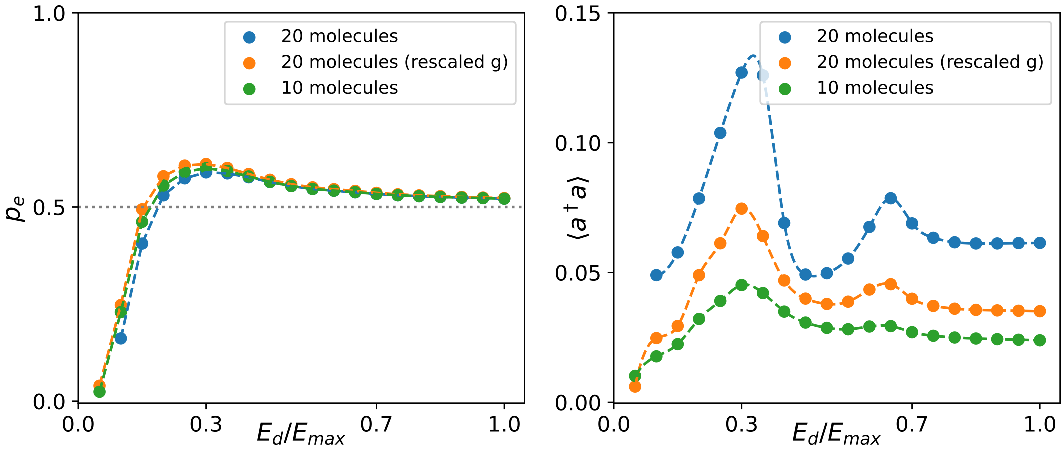


FIG. S3: Electronic inversion and cavity occupation for $N = 10, 20$ molecules from Fig. 2 of the main text compared to a new simulation with $N = 20$ and a rescaled cavity coupling strength $g_{rescaled} = g_{cav}/\sqrt{2}$. Due to the rescaling the collective coupling strength $g_{cav}/\sqrt{N}$ of this new simulation is comparable to the collective coupling strength for $N = 10$ and the original parameters. Nevertheless, the resulting cavity occupation is increased. Dashed lines serve purely as a guide to the eye.

## S2. ADDITIONAL SIMULATION DATA

In the following, we present simulation data for some additional parameters which were not discussed in the main text.

### A. Rescaled coupling strength

The first simulation concerns the enhanced lasing intensity for larger $N$, shown in Fig. 2(c) of the main text. An interesting question is whether this enhancement can be explained by the collectively increased coupling strength $g \to g_{col} = \sqrt{N}g$ typical for the Tavis-Cummings and related models. To answer it we compare the $N = 10, 20$ data shown in Fig. 2 of the main text with an additional simulation for $N = 20$ molecules, were the coupling strength has been rescaled $g_{rescaled} = g_{cav}/\sqrt{2}$. Therefore, the collective coupling strength in this new simulation with $N = 20$ is identical to the collective coupling strength of $N = 10$ molecules for the original parameters (coupling strength $g_{cav} = 0.2E_{max}$). The comparison is shown in Fig. S3 and shows the increase in intensity if the collective coupling $g_{cav}\sqrt{N}$ instead of the individual coupling is kept constant. The intensity still increases with $N$, although less so. This absolute increase in itself is not surprising and can already be obtained at the mean-field level. Perhaps more surprising is that the relative peak heights also increase, which hints towards beyond mean-field effects.

### B. Vibrational occupation

In addition to the data provided in the main text we discuss the occupation of the vibrational modes for $N = 10$ and otherwise identical parameters to the main text. While we can not resolve the individual occupation of each of the 107 vibrational modes due to the use of the effective spectral density $J_{eff}(\omega)$ and HEOM, we are able to reconstruct the occupation in different regions of the spectrum from the HEOM auxiliary states. Below we distinguish between the occupation of the "first peak" corresponding to the region from $\omega = 0$ to $\omega \approx 1E_{max}$ and the "second peak", corresponding to the small maximum in $J_{eff}$ at $\omega \approx 1.3E_{max}$. Fig. S4 compares their occupation for different driving strengths (black curve). The red curve shows the cavity occupation in comparison, which clearly establishes a correlation with the vibrational occupation. The maxima in the vibrational occupation can be explained with the help of $H_{simple} = E_d\sigma_x + g^{vib}\sigma_+\sigma_-(b + b^\dagger) + \omega^{vib}b^\dagger b$ briefly discussed in the main text. In a rotating frame with

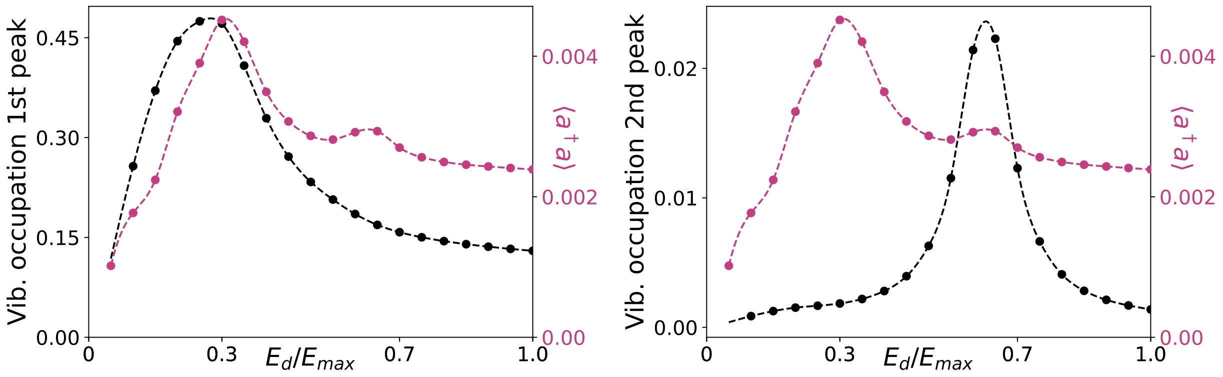


FIG. S4: Vibrational occupation (black dots) over driving strength in different regions of the vibrational spectrum (see text) and $N = 10$. For reference we also show the cavity occupation for the same drive strengths, which highlights a correlation between the cavity and vibrational occupations. Dashed lines serve purely as a guide to the eye.

respect to the drive $\left|\widetilde{\psi}\right\rangle = \exp\left(iE_d\sigma_x + 2E_d b^\dagger b\right)|\psi\rangle$ we find

$$\begin{aligned}\widetilde{H}_{simple} =& (\omega^{vib} - 2E_d)b^\dagger b + g^{vib}(\sigma_{+x}b + \sigma_{-x}b^\dagger) + \frac{g^{vib}}{2}\left(be^{-2iE_dt} + b^\dagger e^{2iE_dt}\right)\\ &+ \frac{g^{vib}}{2}\left(e^{-4iE_dt}b^\dagger\sigma_{+x} + e^{4iE_dt}b\sigma_{-x}\right),\\ \sigma_{+x} =& \frac{\sigma_z - i\sigma_y}{2},\\ \sigma_{-x} =& \frac{\sigma_z + i\sigma_y}{2}.\end{aligned} \tag{S14}$$

Neglecting the rotating terms (valid for $\omega^{vib} \approx 2E_d$), clearly show how the interaction becomes resonant at $\omega_{vib} = 2E_d$, as all other terms vanish. We expect that this increases the population in state 3, i.e., the upper state of the lasing transition, which enhances the lasing and thus populates the cavity mode (magenta curves). A more detailed analytical derivation or studies of suitable minimal models that show this effect is an exiting prospect for future studies.

### C. Off-resonant driving

In the main text we focus on the qualitative features that arise upon changing the driving strength at a fixed frequency, which corresponds to the maximum of the molecular absorption spectrum (without cavity). Fig. S5 shows the behavior of the inversion as well as the vibrational occupation upon varying the drive frequency at a fixed strength $E_d = 0.3E_{max}$. The maximal values are reached close to, but not exactly at $\omega_0$.

## S3. SUMMARY OF THE BBGKY-HEOM METHOD

In the following we give a brief summary of the BBGKY-HEOM method that was used to obtain the reduced dynamics under Eq. (S13) and including the dissipative terms in Eq. (S10). A detailed derivation of the method including several benchmarks can be found in Ref. [8].
The method is based on a combination of the Bogoliubov-Born-Green-Kirkwood-Yvon (BBGKY) hierarchy and the Hierarchical Equations Of Motion (HEOM) and it allows an approximate treatment of many-body systems in contact with both global and local non-Markovian environments. In the present case we treat the electronic states of the molecules as the system and the damped cavity mode as a global bath (meaning it couples equally to every molecule). In addition, the vibrational manifolds form local non-Markovian baths, meaning they couple to only one molecule and there is one such bath per molecule. The core approximation to make the solution of the model tractable is introduced by the BBGKY hierarchy and amounts to a Gaussian approximation of three-particle correlations (and higher). Essentially, we approximate the density matrix of three molecules $\rho_{123}$ in terms of the two-molecule density

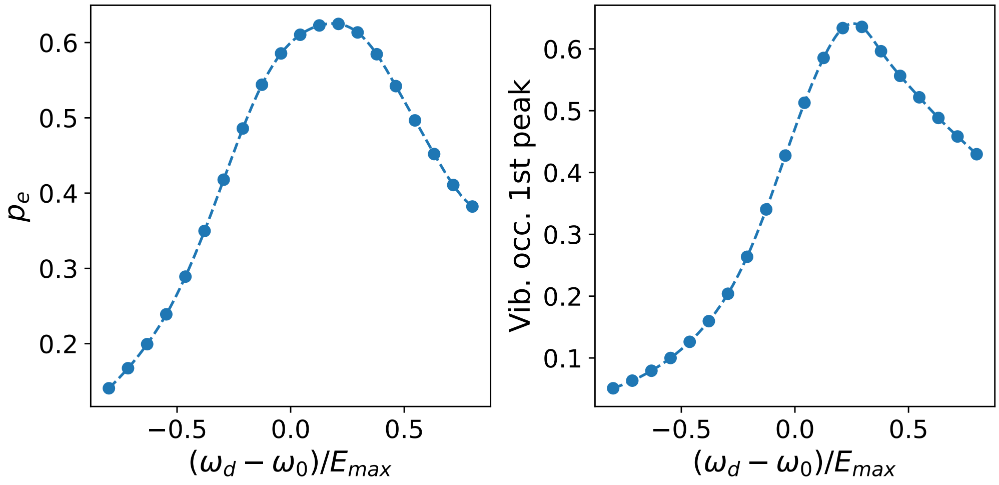


FIG. S5: Inversion and vibrational occupation for different detunings of the drive. Dashed lines serve merely as aguide to the eye.

$\rho_{12}$ and the one molecule density matrix $\rho_1$as

$$\rho_{123} \approx \rho_1\rho_{23} + \rho_{12}\rho_3 + \rho_{13}\rho_2 - 2\rho_1\rho_2\rho_3, \tag{S15}$$

Due to the particle exchange symmetry of our system the reduced one and two particle density matrices $\rho_k$ and $\rho_{ij}$ ($i, j, k \in [1, 2, 3]$) correspond to identical operators, that do however act on different particles. The approximation (S15) allows us to derive a closed equation for the reduced state of two molecules $F_{12}^{(\mathbf{0},\mathbf{0})}(t) = N(N-1)\rho_{12}(t)$, where the normalization is by convention. The HEOM auxiliary states $\rho^{(\boldsymbol{n},\boldsymbol{m})}$ are labeled by the vector indices $\boldsymbol{n}$, $\boldsymbol{m}$ and we denote the unit vector in direction $k$ by $\boldsymbol{e}_k$. The resulting evolution equation reads

$$\begin{aligned}
\dot{F}_{12}^{(\boldsymbol{n},\boldsymbol{m})} =& - i[H_{12}, F_{12}^{(\boldsymbol{n},\boldsymbol{m})}] - (\boldsymbol{w}\cdot\boldsymbol{n} + \boldsymbol{w}^*\cdot\boldsymbol{m})\, F_{12}^{(\boldsymbol{n},\boldsymbol{m})} + g_{cav}\left(n_k(L_{g1}+L_{g2})F_{12}^{(\boldsymbol{n}-\boldsymbol{e}_1,\boldsymbol{m})} + n_k\,\mathrm{tr}_3(L_{g3}F_{123}^{(\boldsymbol{n}-\boldsymbol{e}_1,\boldsymbol{m})})\right) \\
&+ g_{cav}\left(m_k F_{12}^{(\boldsymbol{n},\boldsymbol{m}-\boldsymbol{e}_1)}(L_{g1}^\dagger + L_{g2}^\dagger) + m_k\,\mathrm{tr}_3(L_{g3}^\dagger F_{123}^{(\boldsymbol{n},\boldsymbol{m}-\boldsymbol{e}_1)}) + \left[F_{12}^{(\boldsymbol{n}+\boldsymbol{e}_1,\boldsymbol{m})}, L_{g1}^\dagger + L_{g2}^\dagger\right] + \left[L_{g1}+L_{g2}, F_{12}^{(\boldsymbol{n},\boldsymbol{m}+\boldsymbol{e}_1)}\right]\right) \\
&+ \sum_{k=2}^{2M+1} \sqrt{G_k}\, n_k L_k F_{12}^{(\boldsymbol{n}-\boldsymbol{e}_k,\boldsymbol{m})} + \sqrt{G_k}^*\, m_k F_{12}^{(\boldsymbol{n},\boldsymbol{m}-\boldsymbol{e}_k)} L_k^\dagger + \sqrt{G_k}\left[F_{12}^{(\boldsymbol{n}+\boldsymbol{e}_k,\boldsymbol{m})}, L_k^\dagger\right] + \sqrt{G_k}^*\left[L_k, F_{12}^{(\boldsymbol{n},\boldsymbol{m}+\boldsymbol{e}_k)}\right].
\end{aligned} \tag{S16}$$

We have already adjusted Eq. (S16) to our specific application, where $L_{gj} = \sigma_j^-$ ($j = 1, 2, 3$) is the coupling operator to the global bath (here the cavity mode), $L_k = \sigma_1^+\sigma_1^-$ for even $k$ and $L_k = \sigma_2^+\sigma_2^-$ for odd $k$, which represents the coupling to the local vibrational baths of molecule one and two. The vibrational bath is fitted with $M$ exponentials as $\alpha_{eff}(\tau) = \sum_{k=1}^{M} G_{2k+1} e^{-w_{2k+1}\tau}$. Additionally, we have $G_{2k} = G_{2k+1}$, $w_{2k} = w_{2k+1}$ for $k = 1, ..., k_{max}$ and $w_1 = i\omega_{cav} + \kappa$. Including the hierarchy index the approximation of the three particle matrices in terms of the two particle ones are given as [8]

$$\begin{aligned}
F_{123}^{(\boldsymbol{n},\boldsymbol{m})} =& \, 4\frac{(N-1)(N-2)}{N^3}\,\mathrm{tr}\Big(F_1^{(\boldsymbol{n},\boldsymbol{m})}\Big)F_1F_2F_3 + \frac{N-2}{N}\left(F_{12}F_3^{(\boldsymbol{n},\boldsymbol{m})} + F_2^{(\boldsymbol{n},\boldsymbol{m})}F_{13} + F_1^{(\boldsymbol{n},\boldsymbol{m})}F_{23}\right) \\
&+ \frac{N-2}{N}\left(F_1F_{23}^{(\boldsymbol{n},\boldsymbol{m})} + F_{13}^{(\boldsymbol{n},\boldsymbol{m})}F_2 + F_{12}^{(\boldsymbol{n},\boldsymbol{m})}F_3\right) - \frac{N-2}{N^2}\,\mathrm{tr}\Big(F_1^{(\boldsymbol{n},\boldsymbol{m})}\Big)\left(F_{12}F_3 + F_{13}F_2 + F_1F_{23}\right) \\
&- 2\frac{(N-2)(N-1)}{N^2}\left(F_1^{(\boldsymbol{n},\boldsymbol{m})}F_2F_3 + F_1F_2^{(\boldsymbol{n},\boldsymbol{m})}F_3 + F_1 + F_2F_3^{(\boldsymbol{n},\boldsymbol{m})}\right),
\end{aligned}$$

where $F_j = N\rho_j$.

## S4. NUMERICAL DETAILS

Below we provide additional details on how the simulations shown in Fig. 2 and 3 in the letter were performed. All simulations rely on the fit of the effective bath correlation function shown in Fig. 1(d) in the letter. This fit was

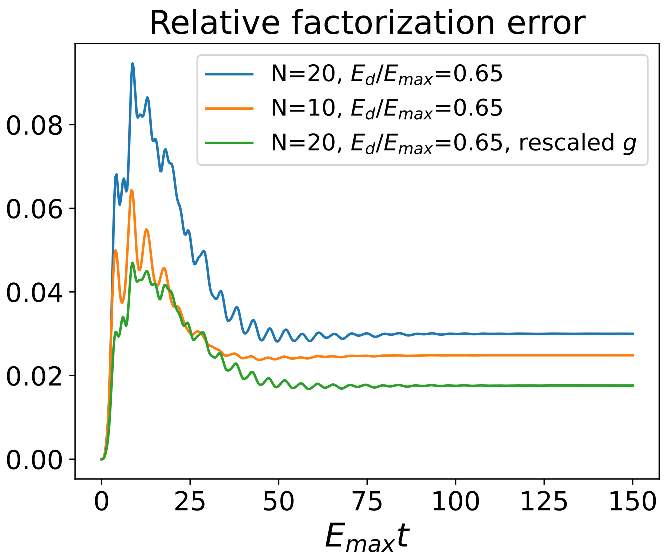


FIG. S6: The relative error of factorising the reduced two-particle density matrix obtained from BBGKY-HEOM as $F_{12}(t) \approx \text{Tr}_2(F_{12}) \otimes \text{Tr}_2(F_{12})$ for $E_d = 0.65E_{max}$. The relative error is obtained based on the Frobenius norm and shown for different atom numbers. The green curve corresponds to a simulation with $N = 20$ molecules and the same collective coupling strength $\sqrt{N}g_{cav}$ as the orange curve, where $N = 10$ molecules were included.

performed using five exponential functions and the optimal parameters were obtained by minimizing the $L^1$ distance over the relevant time interval. To ensure the correctness of our algorithm and Eq. (S8) we checked that an explicit implementation of Eq. (S6) with a *single* vibrational mode (taken into the system) coupled to the ohmic environment $J_E$ agreed with the corresponding BBGKY-HEOM implementation of the effective vibrational bath (S9). For the final simulations we used a hierarchy depth of five for the vibrations and three for the cavity mode. We checked for selected parameters that the results don't change significantly (deviation smaller than the linewidth in the plots) upon increasing the hierarchy depth or when using a fit with 6 exponentials.

Finally, one may ask whether the BBGKY framework is even necessary to investigate this system or whether one might just as well use a mean-field approximation. To answer this question, we compare the reduced electronic state of two molecules obtained from Eq. (S16) $F_{12}(t)$ with its factorized approximation $\text{Tr}_2(F_{12}) \otimes \text{Tr}_2(F_{12})$. The relative error of this approximation is obtained with the help of the Frobenius norm $\| \cdot \|$ as $\|F_{12}(t) - \text{Tr}_2(F_{12}(t)) \otimes \text{Tr}_2(F_{12}(t))\| / \|F_{12}(t)\|$ and shown in Fig. S6 for a driving strength $E_d = 0.65E_{max}$ and different atom numbers. The relative error exceeds 6% for the data discussed in the main text. This would accumulate during time-evolution under a mean-field approximation, highlighting the importance of taking correlations into account. Furthermore, we find that for the parameters considered in the main text ($g_{cav}$ independent of $N$) the relative error increases with the atom number. Rescaling the coupling strength $g \to g/\sqrt{N}$ restores the usual intuition that in all-to-all interacting models mean-field approximations become accurate for large $N$.

[1] F. Neese, Wiley Interdisciplinary Reviews: Computational Molecular Science **12**, e1606 (2022).
[2] J. P. Perdew, K. Burke, and Y. Wang, Physical Review B **54**, 16533 (1996), ISSN 1095-3795.
[3] S. Grimme, J. Antony, S. Ehrlich, and H. Krieg, The Journal of Chemical Physics **132**, 154104 (2010), ISSN 0021-9606.
[4] B. Wolfseder and W. Domcke, Chem. Phys. Lett. **235**, 370 (1995), ISSN 0009-2614.
[5] U. Weiss, *Quantum Dissipative Systems (Third Edition)*, Series In Modern Condensed Matter Physics (World Scientific Publishing Company, 2008), ISBN 9789814471855, URL `https://books.google.de/books?id=p0jICgAAQBAJ`.
[6] J. Roden, W. T. Strunz, K. B. Whaley, and A. Eisfeld, J. Chem. Phys. **137**, 204110 (2012), ISSN 0021-9606.
[7] B. de Souza, G. Farias, F. Neese, and R. Izsák, Journal of Chemical Theory and Computation **15**, 1896 (2019).
[8] K. Müller, K. Luoma, and C. Schäfer (2025), 2405.05093, URL `https://arxiv.org/abs/2405.05093`.
