# Supplementary material for: Vibrationally Induced Resonances in Lasing
Source: J Phys Chem Lett. 2026 Apr 23;17(18):5275–9. doi: 10.1021/acs.jpclett.5c04028 (PMC13158989; doi:10.1021/acs.jpclett.5c04028)
Supplement: Supplementary file 2 [file jz5c04028_si_002.pdf]

jz-2025-04028e.R1

Name: Peer Review Information for "Vibrationally Induced Resonances in Lasing"

First Round of Reviewer Comments

Reviewer: 1

Comments to the Author

In this manuscript, the authors discuss an application of their recently developed BBGKY-HEOM method, presented in an accompanying article, to the problem of few-molecule lasing in plasmonic gap cavities. While I have looked at the accompanying work, it is not my task here to evaluate the method itself. The present manuscript is generally well written, clearly structured, and addresses a relevant gap in the few-emitter cavity regime, where efficient theoretical approaches are currently needed. I have however a few concerns, mainly on the validity of the comparisons, the connection to experiments, as well as on the physical insight provided that I would like the authors to address:

(1) The authors compare coherent and incoherent driving in the plots in Fig 2. using the same rate E_d. I am not sure that a comparison between the two rates makes physical sense, since the effective incoherent drive can actually be derived under the assumption of fast vibrational relaxation from the coherent drive Hamiltonian and in general therefore depends on the molecular parameters (if relaxation rate is gamma it should be something like E_d^2/gamma in the simplest case). If the adiabatic elimination is not valid, it is therefore somewhat expected and not surprising that the incoherent drive approximation fails. As a benchmark check, one should be able to find a regime (e.g., completely white noise unstructured bath) where the two models agree. Moreover, as far as I'm aware, in most cavity-lasing experiments actually not the S_1 first excited electronic state but a higher electronic state is driven, followed by fast non-radiative relaxation to S_1. Then, the incoherent drive stems from the decay from a higher electronic manifold, not from relaxation within the same manifold.

(2) The drive frequency is assumed to be resonant with the Stokes-shifted molecular transition frequency. I find this is a rather restrictive assumption. Can the authors include or at least comment on more general driving frequencies, since vibrational resonances should also appear when scanning the drive frequency?

(3) The authors include linear electron–vibration coupling and a phononic environment dampening the vibrations, but it is known that quadratic electron–vibration coupling can dominate dephasing and lead to strong temperature-dependent linewidth broadening (see, e.g., Phys. Rev. Lett. 124, 153602). Is this temperature dependence included in the present approach? Can the authors e.g. compare their model with an experimentally measured methylene-blue spectrum?

(4) I find the physical interpretation provided in the final part somewhat unsatisfying. In particular, it is surprising that vibrational effects increase with the number of emitters N, I would have expected the opposite. Can the authors comment more on that? In addition, the identification of resonances at 2E_d with peaks in the effective spectral density is not entirely clear to me. While the authors draw an analogy to the quantum Rabi model, the physical interpretation is largely relegated to future studies. Given that JPC Lett places strong emphasis on clear physical insight as a criterion for publication, strengthening this aspect would significantly improve the impact of the work.

some minor points:

- equations extend beyond the page margins and should probably be broken up

- Moore’s law is misspelled in the introduction

- typo (“arise s”) in ln 26 on page 4.

Reviewer: 2

Comments to the Author

I have read the submitted manuscript by Muller et al. along with the supplementary information and the manuscript of paper submitted to Physical Review B (PRB)

The manuscript presents calculations modeling the behavior of a few molecule laser.

The model is of N molecules coupled to a common cavity mode, where each molecule is described by two electronic states dressed by a vibrational continuum. The authors consider coherent driving of the systems, such that decoherence and relaxation of the vibrational state is responsible for the emergence of an effective four-level lasing scenario. This scenario is the standard picture considered for operation of a dye laser, although such descriptions tend to be restricted to the weak light-matter coupling limit. The weak coupling limit of such a model has also been extensively discussed for description of photon condensates. The current work however makes no restriction to weak coupling.

The model system is approximately described by the HEOM-BBGKY approach introduced by the authors in the accompanying PRB. This allows treatment that goes beyond a mean-field description, by including correlations between pairs of molecules.

The results found are compared to a simplified model of incoherent pumped two-level systems. The authors clearly show features that are lacking from that simplified model. In particular, there is a non-monotonic dependence of photon number on pump strength, due to vibrational resonances.

On balance I believe the manuscript does reach the threshold required for publication in the Journal of Physical Chemistry Letters. The method (described in the PRB) is novel and significant, and is used here to address a question of practical relevance. The results found show that there is interesting physics to be explored in this model. The current manuscript does not fully explain all these intriguing results (and instead states that explanation is for a future paper). Considering the JPCL criteria for theory papers this could raise questions about whether this manuscript presents "significant new physical insight". However, combined with the PRB there is a stronger case that this presents "a new theoretical or computational methodology of general interest". As such I would on balance support publication.

I had a few comments about the physics, and a few points on presentation (given below).

* There is other work that has looked at a very similar model (N molecules vibrationally dressed by a structured harmonic bath, coupled to a cavity mode, beyond mean field theory). This is https://arxiv.org/abs/2505.23028 recently accepted at PRL as https://doi.org/10.1103/kjsb-h9s7 . While this other work uses path integral methods rather than HEOM, it uses the same BBGKY/cluster expansion, albeit under a different name. This does not seem to be cited in either the current manuscript or that submitted to PRB. Citing this would seem appropriate.

* While it seems reasonable that the PRB is referenced for details of the derivation of the HEOM-BBGKY approach, it seems odd that this paper (or at least the supplement) do not even contain the basic equation that is used for simulation. I would request the authors to briefly summarize the main equation (equivalent of e.g. Eq. D3 from the PRB) in the supplement, so that this manuscript is self contained in describing what is done.

In particular, one element that is unclear from the model in this paper as compared to the derivation in the PRB. Is the photon mode treated as part of the system, or as an extra environment? Both seem possible, but neither seems described in the PRB. If the photon mode is an extra environment then one needs a version of HEOM-BBGKY that has both a common bath and local baths. If the photon mode is in the system, then not all parts of the system are permutation symmetric (the photon is different from the molecules). Both extensions seem possible, but neither seems to be explicitly presented.

* In figure 2(c), the authors show behavior with increasing N. From the way the model is described it seems that the coupling strength to a single molecule is constant. As such, it is not clear if the main effect here is due to the changing number of molecules, or due to the changing effective coupling g_cav \sqrt{N}.

This raises a related question. While the PRB explicitly considers which behavior is distinct from a mean-field treatment, this is not discussed in this manuscript for the problem studied here. Is such a comparison possible? (That is, a comparison to the HEOM-BBGKY hierarchy but keeping only the one-site density matrices, thus corresponding to a factorized ansatz for the density matrix).

I would expect that for N=5, N=10 results are likely to differ notably from mean field, but this difference may reduce at larger N. There is a general expectation (see e.g. https://arxiv.org/abs/2505.23028 noted above and refs therein) about how mean field should become more accurate at large N. It could be worth showing this comparison.

* In comparing results to a model with incoherent pumping and decay, it was not clear whether this was meant to be purely an indicative comparison to a toy model or something further. If one did try to describe the effect of vibrational modes + driving through effective Markovian rates, I might have expected a model that also had a pure dephasing, which may change the results somewhat. If the Markovian model is meant to be a fair comparison it would seem appropriate to allow pure dephasing, and to discuss further how the rates in that model are derived. However if it is just an example, then the current approach is fine, but should perhaps be clarified.

A few other points of presentation:

* In the abstract there is a confusing sentence: "Utilizing the recently developed stacked hierarchy approach and informed from first principles, we demonstrate the impact of the vibrational structure on lasing at the example of few-molecular lasing in plasmonic cavities." Should this read "Utilizing the recently developed stacked hierarchy approach and informed from first principles, we demonstrate the impact of the vibrational structure on lasing with the example of few-molecule lasing in plasmonic cavities."?

* Opening sentence should read "Moore's law", not "Moors law".

* In the description of Figure 3 the reference to transparent and saturated lines is not clear. It would be clearer to talk about darker/lighter lines, as that is what can be seen on the screen.

Author's Response to Peer Review Comments:

TU Wien, Wiedner Hauptstr. 8-10, 1040 Wien

Christian Schäfer
Wiedner Hauptstr. 8-10
1040 Wien
christian.schaefer@tuwien.ac.at

Editorial Board
JPCL

Vienna, March 31, 2026

Dear Editor, Dear referees,

please find attached our revised manuscript "Vibrationally Induced Resonances in Lasing" by Kai Müller, Kimmo Luoma, and Christian Schäfer.

Furthermore, we provide below a detailed response to all comments and requests. We would like to thank both reviewers for their excellent and constructive reports, which undoubtedly improved the quality of the manuscript.

Pointwise response

Reviewer 1

1. *The authors compare coherent and incoherent driving in the plots in Fig 2. using the same rate E_d. I am not sure that a comparison between the two rates makes physical sense, since the effective incoherent drive can actually be derived under the assumption of fast vibrational relaxation from the coherent drive Hamiltonian and in general therefore depends on the molecular parameters (if relaxation rate is gamma it should be something like E_d^2/γ in the simplest case). If the adiabatic elimination is not valid, it is therefore somewhat expected and not surprising that the incoherent drive approximation fails. As a benchmark check, one should be able to find a regime (e.g., completely white noise unstructured bath) where the two models agree. Moreover, as far as I'm aware, in most cavity-lasing experiments actually not the S_1 first excited electronic state but a higher electronic state is driven, followed by fast non-radiative relaxation to S_1. Then, the incoherent drive stems from the decay from a higher electronic manifold, not from relaxation within the same manifold.*

We completely agree with the reviewer in that the validity of the incoherent drive depends heavily on the molecular characteristics and that there are regimes where we expect this approximation to perform better. However, we focus on the qualitative trend, as stated (originally) in the sentence "We focus our analysis on the qualitative behavior as coherent driving and incoherent pumping will result in different steady-states for a given value." We now emphasize this more by adding the following sentence directly after Equ. 5: "How accurate this approximation is will depend heavily on the coupled bath, i.e., on the molecular and host structure. Additionally, note that this comparison is supposed to be of qualitative nature as the different steady-states for a coherent pumping with strength E_d are not directly comparable to those for incoherent pumping with strength E_d."

Both methods agree in the regime of weak driving (compared to the relaxation timescale), where consequently also the qualitative trends agree in Fig. (2).

Most organic lasers are pumped and operated in the S0-S1 manifold, see e.g. https:// scijournals.onlinelibrary.wiley.com/doi/10.1002/pi.3173. The platform of interest and the corresponding experiment also involve solely the S0-S1 transition. We agree that, in situations where the higher excited states are pumped, fast internal conversion (Kasha's rule) would likely strengthen the incoherent drive approximation. However, the scope of this work is to highlight a previously overlooked feature. A detailed discussion of the limits and validity of the incoherent pump approximation would require a substantially broader study.

2. *The drive frequency is assumed to be resonant with the Stokes-shifted molecular transition frequency. I find this is a rather restrictive assumption. Can the authors include or at least comment on more general driving frequencies, since vibrational resonances should also appear when scanning the drive frequency?*

   In our manuscript we wanted to mainly focus on the perhaps less expected resonances that occur when tuning the driving strength and therefore focused on a fixed driving frequency. This driving frequency was chosen at the maximum of the absorption spectrum of the single molecule (without cavity), which is a reasonable choice and close to the experimental implementation. The referee is correct in pointing out that vibrational resonances appear also when scanning the driving frequency. On a basic

level we discuss this in the context of the absorption spectrum of a single molecule in Fig. S2.

We have now generalized the derivation in Sec. S2 C of the supplemental material to include non-resonant driving and have performed a series of calculations that vary the frequency, while keeping the driving strength fixed at the resonance $E_d = 0.3E_{max}$

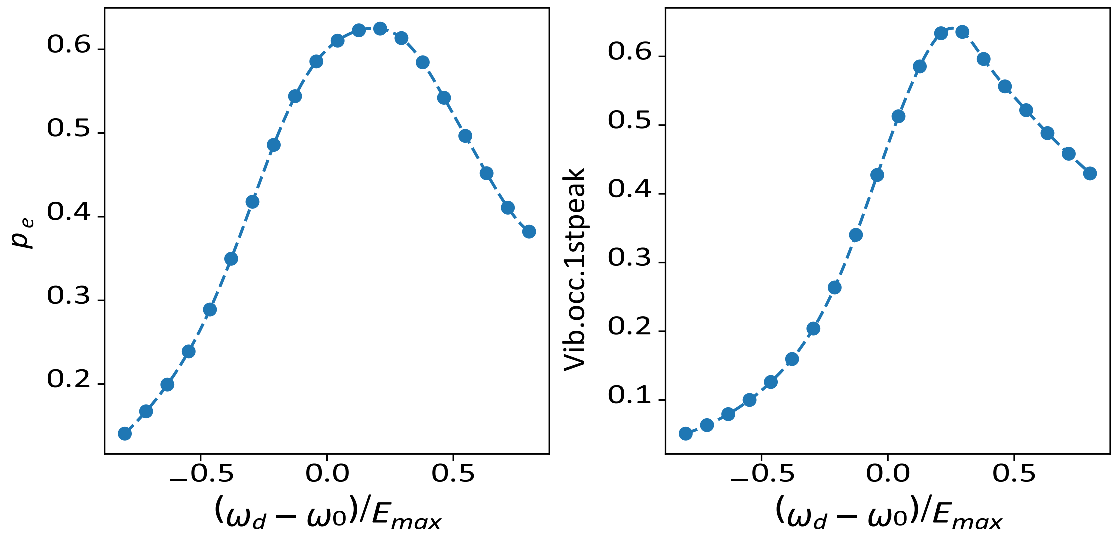

We have included the shown plots and a short discussion in the SI. Future work will further explore the interplay between $E_d, \omega_d, N, g, \omega_{cav}$ and level of multi-emitter correlation.

3. *The authors include linear electron-vibration coupling and a phononic environment dampening the vibrations, but it is known that quadratic electron-vibration coupling can dominate dephasing and lead to strong temperature-dependent linewidth broadening (see, e.g., Phys. Rev. Lett. 124, 153602). Is this temperature dependence included in the present approach? Can the authors e.g. compare their model with an experimentally measured methylene-blue spectrum?*

This is indeed a fascinating question which we expect to play a major role for our future investigations regarding vibrational heating. We fully agree with the reviewer that quadratic contributions, also known as Debye-Waller diagrams, play an important role in temperature dependence. Such terms can be considered with different strategies:

- The Debye-Waller diagrams are quadratic in el.-vib. coupling and therefore usually much smaller than the bilinear terms. In those sitations, a perturbative treatment allows one to recast the quadratic

terms into renormalized bath frequencies and couplings (dependent on T) or into effective dephasing terms.

- Electronic structure approaches and molecular dynamics simulations allows one to create "effective harmonic models" which cast the T-dependence of vibrational or phononic modes into, as the name suggests, purely harmonic models that depend on temperature. [https://doi.org/10.1103/PhysRevB.99.184304] Those effective quantities can be then used to create a T-dependent spectral function for HEOM.
- There exist variations of HEOM, e.g. DEOM [http://arxiv.org/abs/2206.14375v5], in which quadratic coupling can be readily included.
- As pointed out by Tanimura [https://doi.org/10.1063/5.0011599], it is also possible to adjust HEOM, ignoring non-Gaussian features at first, and include them subsequently via non-Gaussian correction (see e.g. https://doi.org/10.1103/ PhysRevLett.109.130401).

In short, it is entirely possible to account for anharmonic coupling effects with manageable additional work, but this extends beyond the scope of the present manuscript.

We show in SI Figure S2 that our model provides very consistent spectra when compared to purely quantum-chemical simulations with the ORCA code. The electronic transition energy of methylene-blue (MB) depends heavily on the environment due to its open shell configuration, i.e., it features a strong solvatochromic effect [https: //www.nature.com/articles/210296a0]. A direct comparison to the experiment would demand therefore a more extensive study on the encapsulation, solvation, and plasmonic screening effects.

4. *I find the physical interpretation provided in the final part somewhat unsatisfying. In particular, it is surprising that vibrational effects increase with the number of emitters N, I would have expected the opposite. Can the authors comment more on that? In addition, the identification of resonances at $2E_d$ with peaks in the effective spectral density is not entirely clear to me. While the authors draw an analogy to the quantum Rabi model, the physical interpretation is largely relegated to future studies. Given that JPC Lett places strong emphasis on clear physical insight as a criterion for publication, strengthening this aspect would significantly improve the impact of the work.*

We agree with the reviewer that our previous explanation was too short, but we are convinced that the revised version provides now a clearer interpretation.

Driving the electronic transition S0-S1 with a continuous laser field $H_{drive} = 2E_d \cos(\omega_0 t)\sum_i \sigma_i^x$ results in an AC Stark-effect, or *classical* Rabi-splitting. Similar to strong coupling in cavities, this results in 2 polaritons (at resonance, ignoring counter-rotating terms) with energies $E_\pm^v \approx \omega_0 + n_v\omega_{vib} \pm E_d$. If the energy of the lower polariton $E_-^{v=1}$ aligns precisely with the energy of the upper polariton $E_+^{v=0}$, a resonant excitation of the corresponding vibrational mode occurs, which facilitates the transfer of energy from state 4 to state 3. This is the case if

$$E_-^{v=1} = E_+^{v=0}, \quad (1)$$

$$\omega_0 + \omega_{vib} - E_d = \omega_0 + E_d, \quad (2)$$

$$\omega_{vib} = 2E_d. \quad (3)$$

We now provide now a more intuitive explanation in the main paper and a more mathematical discussion in the SI. Sec. S2 C includes further numerical evidence. In particular we include a new figure inspecting the occupation of relevant vibrational modes for different E_d (see below). They show clear signs of the resonances and correlate with the lasing intensity.

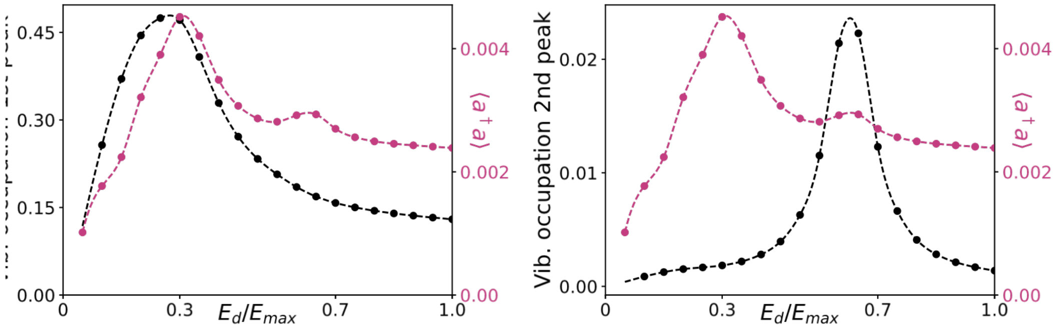

Increased population in state 3, i.e., the upper state of the lasing transition, enhances the lasing and thus populates the cavity mode (magenta curves). Naturally, if we move the frequencies of the relevant vibrations (Fig 3 in the manuscript), the maxima in the lasing curve move. We have revised the corresponding paragraph in the main text to reflect the improved explanation and reference the new section in the SI.

The increasing trend with N depends on 2 components. First, the overall (collective)√ √ coupling to the cavity increases with N. If we compensate for this "trivial" N effect (below, orange line), we can clearly see that the mode-occupation increases less drastically than for the unscaled N = 20 (blue).

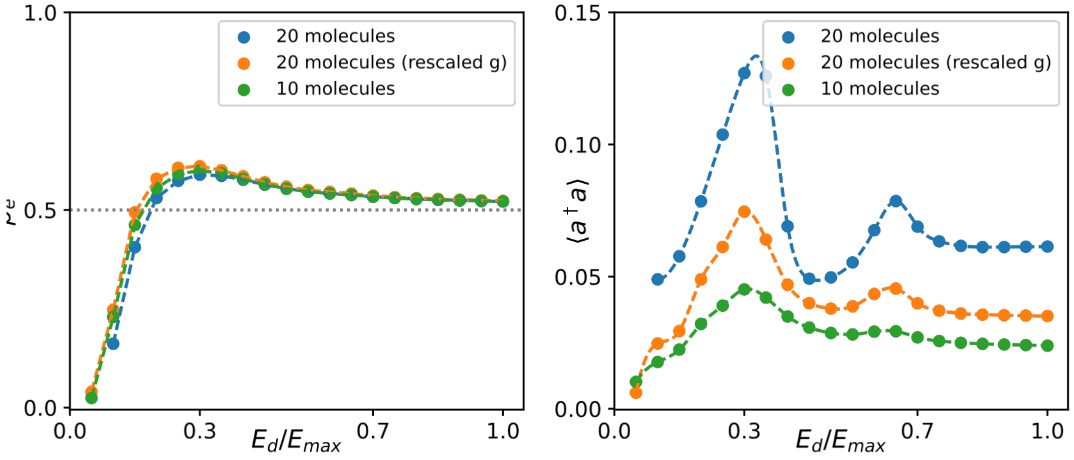

Nevertheless, an increasing number of molecules provides also a larger cross-section for the incoming driving field. More energy is absorbed from the incoming field and emitted to the cavity mode. On a mean-field level one explicitly obtains$\sqrt{\langle a^\dagger a\rangle} \propto N$ for the typical cavity models despite scaling the coupling as g/ N. The absolute increase of the peaks is thus not particularly surprising. More surprising (and perhaps what the referee was solely referring to) is the increased *relative* size of the peak heights. This effect goes beyond the mean-field treatment of typical cavity models and will be explored further in future studies.

Reviewer 2
Major comments

1. *There is other work that has looked at a very similar model (N molecules vibrationally dressed by a structured harmonic bath, coupled to a cavity mode, beyond mean field theory). This is https://arxiv.org/abs/2505.23028 recently accepted at PRL as https://doi.org/10.1103/kjsb-h9s7. While this other work uses path integral methods rather than HEOM, it uses the same BBGKY/cluster expansion, albeit under a different name. This does not seem to be cited in either the current manuscript or that submitted to PRB. Citing this would seem appropriate.*

   We thank the reviewer for raising our awareness for this publication. The reference https://doi.org/10.1103/kjsb-h9s7 has been added to our manuscript that is under revision in PRB. We would like to point out that the first version of our manuscript has been available on arXiv since May 2024, i.e., a full year before https://arxiv. org/abs/2505.23028 has become available on arXiv. Citing our manuscript would thus seem appropriate.

2. *While it seems reasonable that the PRB is referenced for details of the derivation of the HEOM-BBGKY approach, it seems odd that this paper (or at least the supplement) do not even contain the basic equation that is used for simulation. I would request the authors to briefly summarize the main equation (equivalent of e.g. Eq. D3 from the PRB) in the supplement, so that this manuscript is self contained in describing what is done. In particular, one element that is unclear from the model in this paper as compared to the derivation in the PRB. Is the photon mode treated as part of the system, or as an extra environment? Both seem possible, but neither seems described in the PRB. If the photon mode is an extra environment then one needs a version of HEOM-BBGKY that has both a common bath and local baths. If the photon mode is in the system, then not all parts of the system are permutation symmetric (the photon is different from the molecules). Both extensions seem possible, but neither seems to be explicitly presented.*

   We agree with the reviewer and have included a short overview to the methodology to the SI in Sec. S3. It also contains the explicit evolution equation - with both a common (photon) and local (vibrational) baths - obtained from BBGKY-HEOM.

3. *In figure 2(c), the authors show behavior with increasing N. From the way the model is described it seems that the coupling strength to a single molecule is constant. As such, it is not clear if the main effect here is due to the changing number of molecules, or due√ to the changing effective coupling g_{cav} N.*

   We thank the reviewer for this question and agree that the following answer assists the transfer of physical insight.

   √

   If we compensate for the "trivial" *N* effect (below, orange line), we can clearly see that the mode occupation increases less drastically with N.

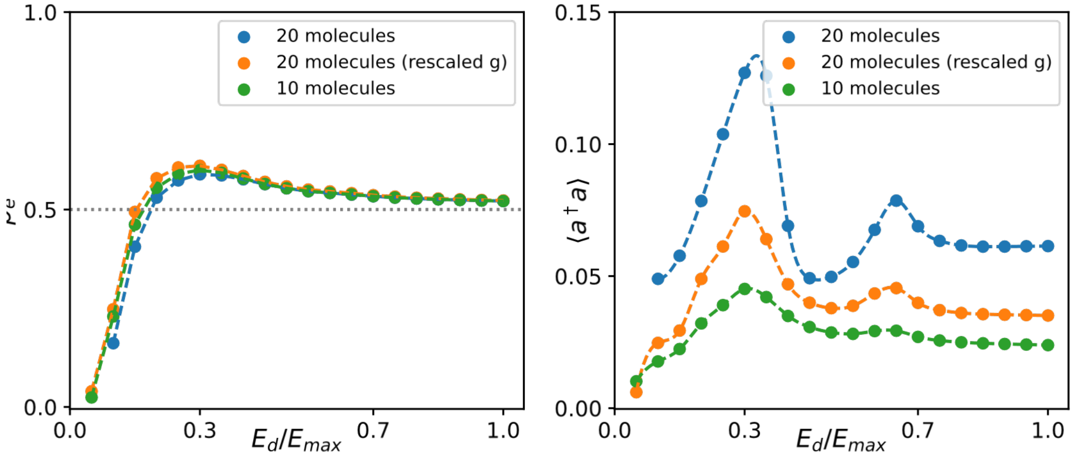

As demonstrated by the orange curve, the increased occupation, and with it the resonant features, are not merely a trivial feature of the collective coupling strength. This in itself is not particularly surprising, as increasing N also increases the optical cross-section of the molecular ensemble, thus more energy is absorbed from an incoming field and emitted to the cavity mode. For example, on a mean field level one explicitly obtains$\sqrt{\langle a^\dagger a\rangle} \propto N$ for the typical cavity models despite scaling the coupling as g/ N. Perhaps more surprising is, that beyond these mean-field effects our results show that the relative height of the vibrational peaks also increases with N.

It should be noted that in the referenced experiments, the quantization volume (and thus g) is fixed, and that increasing N will naturally increase the collective/effective coupling strength.

We have revised our discussion on page 4 and 5 to reflect those insights.

4. *This raises a related question. While the PRB explicitly considers which behavior is distinct from a mean-field treatment, this is not discussed in this manuscript for the problem studied here. Is such a comparison possible? (That is, a comparison to the HEOM-BBGKY hierarchy but keeping only the one-site density matrices, thus corresponding to a factorized ansatz for the density matrix). I would expect that for N=5, N=10 results are likely to differ notably from mean field, but this difference may reduce at larger N. There is a general expectation (see e.g. https://arxiv.org/abs/2505.23028 noted above and refs therein) about how mean field should become more accurate at large N. It could be worth showing this comparison.*

   We agree that such a comparison would add interesting insights to the present study. Instead of propagating a factorized Ansatz we check to which degree the solution we obtain from BBGKY-HEOM factorizes.

Specifically, we compare in the SI Sec. S4 the reduced electronic state of two molecules $F_{12}(t)$ with its factorized approximation $\mathrm{Tr}_2(F_{12})\otimes\mathrm{Tr}_2(F_{12})$. The relative error of this approximation is obtained with the help of the Frobenius norm $\|\cdot\|$ as $\|F_{12}(t)-\mathrm{Tr}_2(F_{12}(t))\otimes \mathrm{Tr}_2(F_{12}(t))\|/\|F_{12}(t)\|$ and shown in a new figure (see below) for one representative driving strength and different atom numbers. We find that for the parameters considered in the main text (g_{cav} independent of N) the relative error in fact increases with the atom√ number. However, rescaling the coupling strength $g \to g/ N$ restores the general expectation that mean-field approximations in all-to-all interacting models become valid for larger N.

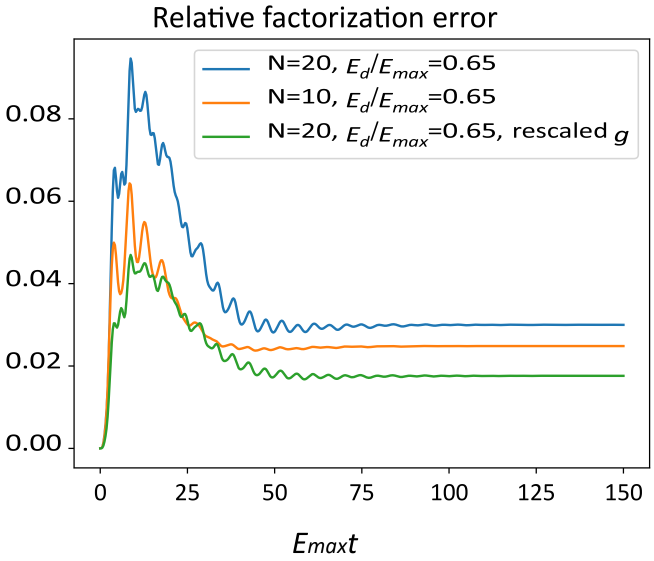

5. *In comparing results to a model with incoherent pumping and decay, it was not clear whether this was meant to be purely an indicative comparison to a toy model or something further. If one did try to describe the effect of vibrational modes + driving through effective Markovian rates, I might have expected a model that also had a pure dephasing, which may change the results somewhat. If the Markovian model is meant to be a fair comparison it would seem appropriate to allow pure dephasing, and to discuss further how the rates in that model are derived. However if it is just an example, then the current approach is fine, but should perhaps be clarified.*

In the specific case of resonant driving considered here one would in fact not obtain additional pure dephasing terms on top of the incoherent pumping and decay (see for example Eq. (3), where the coupling to the vibrational modes does not commute with the system Hamiltonian). Nevertheless, the comparison with the incoherent pumping is supposed to

be a qualitative/indicative one. We have now clarified this by adding the sentence: "Note that this comparison is supposed to be of qualitative nature as the different steady-states for a coherent pumping with strength E_d are not directly comparable to those for incoherent pumping with strength E_d." For a quantitatively exact, ab-initio modeling of the experiment additional dephasing effects would surely need to be included. However, they would need to be added to both models.

Minor comments

1. *In the abstract there is a confusing sentence: "Utilizing the recently developed stacked hierarchy approach and informed from first principles, we demonstrate the impact of the vibrational structure on lasing at the example of few-molecular lasing in plasmonic cavities." Should this read "Utilizing the recently developed stacked hierarchy approach and informed from first principles, we demonstrate the impact of the vibrational structure on lasing with the example of few-molecule lasing in plasmonic cavities."?*

   We thank the reviewer for catching this confusing statement. We have now reformulated the sentence: "Utilizing the recently developed stacked hierarchy approach, informed by first principles, we demonstrate the impact of vibrational structure on lasing, using the example of few-molecule lasing in plasmonic cavities".

2. *In the description of Figure 3 the reference to transparent and saturated lines is not clear. It would be clearer to talk about darker/lighter lines, as that is what can be seen on the screen.*

   We refer now also to full, dashed, and dotted lines to avoid confusion.

We thank the reviewers for their excellent and constructive reports. The revised version is notably improved by the reviewers effort.

Yours sincerely,

Kai Müller and Christian Schäfer, on behalf of all authors.
